# Tri‐trophic interactions: bridging species, communities and ecosystems

**DOI:** 10.1111/ele.13392

**Published:** 2019-10-21

**Authors:** Luis Abdala‐Roberts, Adriana Puentes, Deborah L. Finke, Robert J. Marquis, Marta Montserrat, Erik H. Poelman, Sergio Rasmann, Arnaud Sentis, Nicole M. van Dam, Gina Wimp, Kailen Mooney, Christer Björkman

**Affiliations:** ^1^ Departamento de Ecología Tropical Campus de Ciencias Biológicas y Agropecuarias Universidad Autónoma de Yucatán Km. 15.5 Carretera Mérida‐Xmatkuil MX‐97000 Mérida Yucatán México; ^2^ Department of Ecology Swedish University of Agricultural Sciences Box 7044 SE‐750 07 Uppsala Sweden; ^3^ Division of Plant Sciences University of Missouri 1‐33 Agriculture Building US‐65211 Columbia MO USA; ^4^ Department of Biology and the Whitney R. Harris World Ecology Center University of Missouri–St. Louis 1 University Boulevard US‐63121 St. Louis MO USA; ^5^ Instituto de Hortofruticultura Subtropical y Mediterránea “La Mayora” (IHSM‐UMA‐CSIC) Consejo Superior de Investigaciones Científicas E‐29750 Algarrobo‐Costa (Málaga) Spain; ^6^ Laboratory of Entomology Wageningen University P.O. Box 16 6700 AA Wageningen The Netherlands; ^7^ Institute of Biology University of Neuchâtel Rue Emile‐Argand 11 CH‐2000 Neuchâtel Switzerland; ^8^ UMR RECOVER IRSTEA Aix Marseille University 3275 route Cézanne 13182 Aix‐en‐Provence France; ^9^ Molecular Interaction Ecology Friedrich‐Schiller‐University Jena & German Centre for Integrative Biodiversity Research (iDiv) Halle‐Jena‐Leipzig Deutscher Platz 5e DE‐04103 Leipzig Germany; ^10^ Department of Biology Georgetown University 406 Reiss Science Building US‐20057 Washington DC USA; ^11^ Department of Ecology and Evolutionary Biology University of California Irvine 321 Steinhaus Hall US‐92697 Irvine CA USA

**Keywords:** abiotic forcing, arthropod behaviour, ecosystem effects, food web, indirect defence, trophic cascade

## Abstract

A vast body of research demonstrates that many ecological and evolutionary processes can only be understood from a tri‐trophic viewpoint, that is, one that moves beyond the pairwise interactions of neighbouring trophic levels to consider the emergent features of interactions among multiple trophic levels. Despite its unifying potential, tri‐trophic research has been fragmented, following two distinct paths. One has focused on the population biology and evolutionary ecology of simple food chains of interacting species. The other has focused on bottom‐up and top‐down controls over the distribution of biomass across trophic levels and other ecosystem‐level variables. Here, we propose pathways to bridge these two long‐standing perspectives. We argue that an expanded theory of tri‐trophic interactions (TTIs) can unify our understanding of biological processes across scales and levels of organisation, ranging from species evolution and pairwise interactions to community structure and ecosystem function. To do so requires addressing how community structure and ecosystem function arise as emergent properties of component TTIs, and, in turn, how species traits and TTIs are shaped by the ecosystem processes and the abiotic environment in which they are embedded. We conclude that novel insights will come from applying tri‐trophic theory systematically across all levels of biological organisation.

## Graphical abstract

Tri‐trophic research has followed two distinct paths: One has focused on the population biology and evolutionary ecology of simple food chains of interacting species; the other has focused on bottom‐up and top‐down controls over the distribution of biomass across trophic levels and ecosystem‐level variables. Here, we provide a roadmap to bridge these two long‐standing perspectives and identify two key challenges. First, determining whether and how ecosystem‐level TTIs emerge from food chain‐ and community‐level TTIs. Second, determining whether and how ecosystem‐level processes and abiotic factors feedback to shape the species traits that drive TTIs.

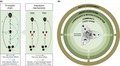

## Introduction

Ecological and evolutionary outcomes of species interactions can only be fully understood after considering the multi‐trophic setting in which species are embedded. For example, phytophagous insects in terrestrial ecosystems go through periodic outbreaks in North America and Europe, destroying millions of hectares of forest each year (McManus *et al.* 1992; Li *et al.* 2015). These outbreaks are often driven by both the loss of natural enemies (parasitoids, predators or pathogens), which would otherwise keep herbivore populations in check (Turchin *et al.* 1999), as well as by changes in host plant resistance and nutritional quality (Turchin *et al.* 1991). Similarly, highly damaging algal blooms in aquatic systems worldwide are driven both by increases in algal resources from eutrophication, as well as by natural enemy suppression of herbivorous zooplankton that otherwise would regulate algal density (Carpenter *et al.* 1985; Micheli 1999). But critically, the effects of these multiple drivers can also interact, resulting in emergent properties across multiple trophic levels that cannot be predicted from separately analysing each component pairwise interaction; for example, tree defences can alter predation or parasitism of herbivores by reducing herbivore performance (Elderd *et al.* 2013), whereas nutrient‐driven algal blooms can lengthen food chains that feedback to increase predator top‐down control (Oksanen *et al.* 1981; Power 1990). Accordingly, these and numerous other so‐called tri‐trophic interactions (TTIs) determine the population biology and evolutionary dynamics of species at all trophic levels and drive fundamental aspects of community structure and ecosystem dynamics. By improving our understanding of ecological and evolutionary processes, tri‐trophic research provides opportunities to establish linkages across levels of biological organisation.

Despite its unifying potential, research on TTIs has been fragmented, following two distinct paths. On the one hand, researchers have adopted a ‘species interactions perspective’, focusing on the evolutionary ecology and population biology of simple food chains of interacting species consisting of herbivore and natural enemy species or guilds associated with one or a few plant species (e.g. Price *et al.* 1980; Mooney & Singer 2012). Concurrently, a separate ‘ecosystem perspective’ on TTIs has focused on the bottom‐up (resource) and top‐down (consumer) controls over the distribution of biomass across trophic levels and other ecosystem‐level properties (e.g. Polis 1999; Borer *et al.* 2005; Hillebrand *et al.* 2007). Bridging these levels of organisation is not only a grand challenge but also a fundamental requirement for developing a full and predictive understanding of community and ecosystem functioning.

We argue that an expanded theory on TTIs can unify our understanding of biological processes across levels of organisation, from species interactions and evolution within simple food chains, to community structure and ecosystem function. Although tri‐trophic research has been previously summarised within distinct subfields (see Box 1), no broad synthesis across all facets of research on TTIs has been offered. Here, we first review the history of research within the species interactions and ecosystem perspectives. Second, we point at gaps in tri‐trophic research within each level of organisation and identify promising opportunities to bridge focal interactions and ecosystem processes, including the application of new technologies and data sources. A common challenge to all of biology (and science) is to link processes across scales and levels of organisation. We hereby argue that a tri‐trophic framework is necessary to address such a challenge in ecology and evolutionary biology.

Box 1 **Previously reviewed sub‐fields of research on tri‐trophic interactions**

The study of TTIs reaches back more than four decades and has been synthesised by reviews focusing on two separate perspectives, one studying population dynamics and evolutionary ecology of species interactions in simple linear food chains and the other on ecosystem‐level processes. A comprehensive synthesis across topics within each perspective or across perspectives has not yet been offered. Below, we provide representative examples of syntheses within sub‐fields of each perspective.

*Species interactions perspective*

- Plant effects on herbivore development time influencing susceptibility to predation (i.e. ‘Slow‐Growth, High‐Mortality Hypothesis’; e.g. Williams 1999).
- Dual effects of plants and natural enemies on herbivore behaviour and evolution (i.e. ‘Enemy Free Space Hypothesis’ and ‘Physiological Efficiency Hypothesis’; e.g. Singer & Stireman 2005; Mooney *et al.* 2012; Vidal & Murphy 2018).
- Plant indirect defences from traits attracting natural enemies that reduce herbivory (e.g. Kessler & Heil 2011; Turlings & Erb 2018; Pearse *et al*. in review).
- Trophic cascades involving natural enemy‐induced changes in herbivore behaviour (Preisser *et al.* 2005).
- Indirect evolutionary effects of natural enemies on lower trophic levels, including natural enemy indirect effects on plant fitness (Romero & Koricheva 2011) and non‐additive selection in tri‐trophic systems (Estes *et al.* 2013; Abdala‐Roberts & Mooney 2015).
- Elevational gradients in natural enemy effects and plant indirect defences (Moreira *et al.* 2018b).
- Plant diversity effects on natural enemy abundance and diversity and its feedback on lower trophic levels (e.g. Letourneau *et al.* 2011; Moreira *et al.* 2016).

*Ecosystem‐level perspective*

- Ecosystem‐level trophic cascades in aquatic or terrestrial systems involving vertebrate predators (e.g. Strong 1992; Pace *et al.* 1999; Shurin *et al.* 2002; Mooney *et al.* 2010; Estes *et al.* 2011; Dirzo *et al.* 2014; Sydeman *et al.* 2015).
- Trophic cascades involving vertebrate and/or invertebrate natural enemies in terrestrial communities (e.g. Schmitz *et al.* 2000; Halaj & Wise 2001).
- Ecosystem consequences of trophic cascades involving predator effects on herbivore behaviour (e.g. Ripple & Beschta 2004; Schmitz *et al.* 2004).
- Effects of consumers and resources on biomass distribution across trophic levels (e.g. Borer *et al.* 2006; Gruner *et al.* 2008).

## Overview of Tri‐Trophic Research

### Species interactions perspective

A vast amount of research on TTIs from a ‘species interactions perspective’ has focused on food chains of one plant species and an associated herbivore and natural enemy species (or guild). Early research treated pairwise interactions among trophic levels as constants and assumed that multi‐trophic systems could be understood by stringing together these pairwise interactions in an additive fashion (e.g. Rosenzweig 1973). The review by Price *et al. *(1980) represented a fundamental turning point, suggesting that pairwise interactions in multi‐trophic systems were in fact inter‐dependent. These authors discussed ways in which plant traits alter herbivore–natural enemy interactions from a population biology and evolutionary ecology standpoint. Their seminal work marked the beginning of considering trophic level inter‐dependence and emergent non‐additive properties stemming from multi‐trophic interactions. The studies that followed applied the Price *et al*. model to all subsets of interactions within tri‐trophic food chains, namely, natural enemy effects on herbivore–plant interactions, herbivore effects on plant–natural enemy interactions, and plant effects on herbivore–natural enemy interactions. This framework was then also applied to belowground TTIs (e.g. Rasmann *et al.* 2005), with a focus on the linkages between above and belowground interactions via plant traits mediating direct and indirect defence (van Dam 2012), as well as research investigating how plant genetic variation underlies these TTIs (Mooney & Singer 2012). The basic concept of non‐additivity has therefore been expanded to all component pairwise interactions in tri‐trophic systems (see Box 2), leading to the development of many complementary and overlapping ecological and evolutionary theories that have fleshed out the details of the causes and consequences of non‐additive interactions (see Mooney & Singer 2012).

Box 2 **Non‐additive tri‐trophic interactions from two perspectives**

A fundamental feature of tri‐trophic systems is that interactions between two trophic levels can be modified by a third trophic level and therefore lead to non‐additive outcomes that cannot be predicted on the basis of pairwise interactions between trophic levels. The study of pairwise interactions in isolation may therefore lead to erroneous conclusions about the nature and consequences of multi‐species interactions. These non‐additive dynamics have been considered from both the species interaction and ecosystem perspectives in fundamentally different ways.

*Species interactions perspective*

Under this perspective, non‐additive effects can be broadly classified into three types of interactions, each of which highlights diverse phenomena and are common to any type of tri‐trophic food chain:

- *Natural enemies alter plant–herbivore interactions*. Natural enemies reduce herbivore abundance and affect herbivore traits (e.g. morphology, behaviour) and, in doing so, indirectly influence patterns and the amount of herbivory. These indirect effects alter plant trait evolution, population dynamics, and community structure. Natural enemies may also directly influence plant traits (e.g. rewards, cues produced by plants in the presence of predators) and this can affect plant relative allocation to direct vs. indirect defences and in turn herbivores.
- *Plants alter herbivore–natural enemy interactions*. Plant traits (e.g. nutrients, secondary metabolites) influence herbivores, which indirectly affects natural enemies. Plants may directly influence natural enemies through the production of cues (volatile organic compounds), rewards (food) or morphological (e.g. shelter in domatia, plant architecture) traits to alter natural enemy behaviours in ways that reduce or enhance herbivory.
- *Herbivores alter plant–natural enemy interactions*. Natural enemy indirect effects on plants are contingent on herbivore traits influencing risk of predation or parasitism (e.g. chemical and behavioural defences). Conversely, the expression of plant traits that attract predators or parasitoids is contingent upon the presence, type, and amount of herbivory via plant‐induced responses to damage.

*Ecosystem perspective*

Research on TTIs from the ecosystem perspective has considered non‐additive effects in three separate contexts.

- *Bottom‐up and top‐down control*. Feedbacks between the bottom‐up effect of plant productivity and the top‐down effects of natural enemies, where increasing productivity increases top‐down control though higher natural enemy density but may also reduce top‐down control by extending food chain length, resulting in secondary predators or parasitoids suppressing primary predators or parasitoids.
- *Plant community composition.* Increased herbivory following reduction in predation and parasitism (i.e. trophic cascades) leads to changes in plant communities from herbivore tolerant to herbivore resistant species, thus altering plant–herbivore interactions.
- *Herbivore behaviour.* Natural enemies induce changes in herbivore behaviours through plastic responses or shifts in species composition that reduce herbivory.

**Figure 1:**
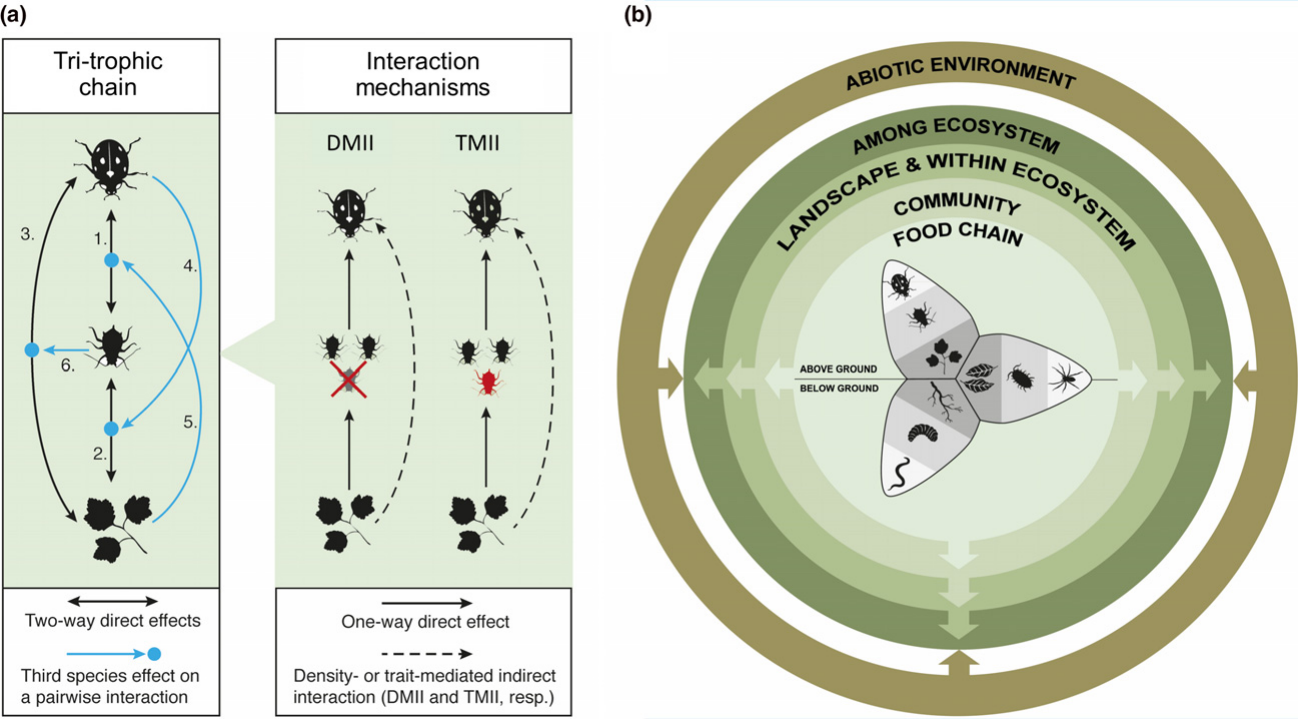
(a) Mechanisms underlying simple food chain TTIs. *Left‐side diagram*: species interactions within a tri‐trophic system illustrated by a species of plant, insect herbivore and invertebrate natural enemy. Effects stemming from pairwise interactions (direct effects) are indicated by solid black arrows, whereas effects involving all three trophic levels are shown in blue and describe non‐additivity for which a species in a given trophic level alters the pairwise interaction between species at the other two trophic levels. Specifically, arrows 1 and 2 indicate two‐way direct effects stemming from pairwise interactions between species at adjacent trophic levels, and arrow 3 depicts two‐way direct effects between the plant and the natural enemy. The remaining (blue) arrows indicate interactions involving species at all three trophic levels potentially leading to non‐additive outcomes: arrow 4 denotes effects of the natural enemy on the plant–herbivore interaction, arrow 5 effects of the plant on the herbivore–natural enemy interaction, and arrow 6 effects of the herbivore on the plant–natural enemy interaction. Blue dots denote a change in each of these pairwise interactions due to the third species leading to non‐additive outcomes (see main text section 2, ‘interactions perspective’). *Right‐side diagram*: Effects of a third species on the pairwise interaction between the other two (arrows 4–6; see examples in Box 2) involve indirect effects which may be density‐ or trait‐mediated and are depicted by broken blue arrows. Shown are a density‐mediated indirect interaction (DMII) where the plant influences herbivore density (depicted as a reduction in herbivore density, e.g. due to plant‐induced resistance), which indirectly influences natural enemy density, and a trait‐mediated indirect interaction (TMII) where the plant influences herbivore traits (change in herbivore colour indicating trait change) and this indirectly affects the natural enemy. TMIIs (but not DMIIs) invariably lead to non‐additive effects. Other DMIIs and TMIIs not shown (for simplicity) include effects of the plant on natural enemy traits or density leading to an indirect effect on the herbivore, effects of the herbivore on plant traits or density indirectly influencing the natural enemy, and effects of the natural enemy on herbivore traits or density indirectly affecting plants. (b) Tri‐trophic food chains are immersed within a broader ecological context. Component TTIs unfold in above‐ and belowground settings where living plants (‘green’ food webs occurring in above‐ or belowground ‘channels’) or detritus (‘brown’ webs occurring in the detritivore ‘channel’) are the basal resource. This broader context involves changes in community structure, landscape‐level dynamics and ecosystem properties that emerge from component TTIs, as well as abiotic variation shaping ecosystem properties which feeds back to affect component food chains. In this way, TTIs outwardly affect the broader ecological context in which they are embedded and at the same time respond to such abiotic context.

From an ecological standpoint, TTIs have been studied within the context of indirect interactions mediated by changes in both the density and traits (i.e. plasticity) of trophically intermediate species (Ohgushi *et al.* 2012). The so‐called density‐mediated indirect interactions (DMIIs) occur where indirect effects are transmitted by changes in the density (not traits) of the intermediate species (Fig. 1a). In contrast, trait‐mediated indirect interactions (TMIIs) are transmitted by changes in the traits of intermediate species (Fig. 1a). Well‐studied examples include natural enemy‐induced changes in herbivore behaviour that indirectly affect plants (Preisser *et al.* 2005) and herbivore‐induced changes in plant traits that influence natural enemy behaviour (e.g. herbivore‐induced plant volatiles ‘HIPVs’; Turlings *et al.* 1990; Kessler & Heil 2011). These and other types of tri‐trophic TMIIs can be classified based upon the trophic level for which traits change, the trophic level inducing those changes, and the trophic level being affected by those changes. Where the strength of TMIIs and DMIIs has been compared, they have been found to be of roughly equal magnitude (Preisser *et al.* 2005), and within the same system they may act concurrently and influence each other (Griffin & Thaler 2006). These ecological dynamics, in turn, have implications for species evolution within tri‐trophic food chains, and the distinction between DMIIs and TMIIs is important in this regard since only the latter are proposed to alter natural selection on species traits (Inouye & Stinchcombe 2001; Abdala‐Roberts & Mooney 2015).

Several overlapping theories have been developed on plant and herbivore evolution within a tri‐trophic context (Mooney *et al.* 2012). Sub‐lethal plant defences have been proposed to mediate herbivore susceptibility to natural enemies by slowing development during vulnerable life stages (Slow Growth‐High Mortality Hypothesis; Moran & Hamilton 1980; Clancy & Price 1987). Similarly, plant indirect defence occurs when plant traits reduce herbivory and increase fitness by boosting the abundance and attack rate of natural enemies (Heil 2008; Kessler & Heil 2011; Pearse *et al*. in review). The adaptive role of indirect defence traits has been implied in many plant‐arthropod systems (Dicke & Baldwin 2010; Hare 2011), including plant traits that provide resources (e.g. extra‐floral nectar) or refuge (e.g. domatia) to natural enemies (Rico‐Gray & Oliveira 2007), as well as information on prey presence (e.g. HIPVs; Kessler & Heil 2011).

From the herbivore’s perspective, the adaptive value of a narrow diet breadth has been studied with respect to the opportunity that it provides enemy‐free space through superior crypsis or sequestration of plant toxins (Enemy‐Free Space Hypothesis; Bernays 1988; Bernays & Graham 1988). Such cases are frequently observed for specialists feeding on toxic plants; these herbivores usually have greater resistance against parasitoids or predatory arthropods (e.g. Petschenka & Agrawal 2016) and pathogens (e.g. Barthel *et al.* 2016). At the same time, herbivores with broad diet breadth can self‐medicate to provide resistance against more specialised natural enemies including parasitoids (Singer *et al.* 2004) and pathogens (Gassman *et al.* 2010). These complementary hypotheses on plant and herbivore evolution have been consolidated within the Tri‐Trophic Interactions Hypothesis, which considers the non‐additive, combined effects of the dynamics predicted by the SGHM, EFS, and other theories (Mooney *et al.* 2012).

In contrast to herbivores and plants, little tri‐trophic theory has been developed from the perspective of natural enemy evolution. Parasitoid‐ or predator‐avoidance traits in herbivores (e.g. crypsis, concealed feeding) have clearly selected for the sophisticated traits used by predators to locate these prey (Abrams 2000). Similarly, plants have also presumably selected for natural enemy traits or behaviours that enhance deterrence or consumption of herbivores (e.g. ant aggressiveness; Rico‐Gray & Oliveira 2007). Finally, natural enemies probably also evolve in response to the dual influences of herbivores and plants in the context of herbivore crypsis, the construction of shelters on plants by herbivores (Lill & Marquis 2007), and sequestration of plant toxins by herbivores (Singer *et al.* 2014).

### Ecosystem perspective

Parallel to the species interactions perspective, a separate ‘ecosystem perspective’ on TTIs has also developed. Ecosystem‐level TTIs include processes underlying the distribution of biomass among trophic levels, as well as the direct and indirect effects of TTIs on ecosystem‐level processes (e.g. nutrient cycles, decomposition rates). Trophic cascades occur from the top‐down when natural enemies indirectly control plant biomass (Polis *et al.* 2000; Shurin *et al.* 2002; Estes *et al.* 2011), from the bottom‐up when resource availability indirectly controls natural enemy biomass (Borer *et al.* 2006; Hanley & La Pierre 2015), and these two dynamics may also interact within (Polis 1999; Borer *et al.* 2005; Hillebrand *et al.* 2007) and across (Knight *et al.* 2005) ecosystems (Box 1).

Early theoretical models that stimulated ecosystem‐level tri‐trophic research were put forward by Fretwell (1977) and Oksanen *et al. *(1981), who extended the ideas of Hairston *et al. *(1960) by proposing that the number of trophic levels increases with ecosystem primary productivity. They argued that systems with four trophic levels are not ‘green’ (i.e. have lower standing stocks of plant biomass) because secondary predators reduce predation pressure on herbivores by preying on primary predators, thereby increasing herbivory. The first empirical studies of ecosystem‐level TTIs came from work on trophic cascades in aquatic ecosystems (e.g. Paine 1969; Estes & Palmisiano 1974; Carpenter *et al.* 1987). Interestingly, most of this research developed independently of the early theoretical models of trophic control in terrestrial ecosystems developed by Hairston *et al. *(1960), Fretwell (1977) and Oksanen *et al. *(1981), although these traditions subsequently converged (e.g. Power 1990).

Drawing on the models of Fretwell and Oksanen *et al.*, top‐down predator effects were compared to bottom‐up effects of primary productivity, and how these factors interacted to determine food chain length, the distribution of biomass across trophic levels, and variation in such patterns among ecosystems (Strong 1992; Pace *et al.* 1999; Borer *et al.* 2005; Borer *et al.* 2006; Hillebrand *et al.* 2007). Bottom‐up and top‐down effects were typically compared with respect to their individual strengths. To the extent that they were proposed to interact, the mechanisms for non‐additivity were focused mostly on variation in abundance, biomass, or diversity of whole trophic levels or guilds (e.g. Duffy 2003; Schmitz 2006), rather than the abundances of individual species taking part in TTIs (see Box 2). In addition, these studies considered TMIIs mainly with respect to changes in plant community composition, either between herbivore‐resistant and tolerant communities (Hanley & La Pierre 2015), or for natural enemy‐induced behavioural changes in herbivores (Schmitz *et al.* 2004; Ripple & Beschta 2004). Although this body of research addressed the mechanisms underlying changes in top‐down vs. bottom‐up control, studies usually involved measurements or manipulations of entire trophic levels or guilds. In cases where effects of individual species were considered (e.g. when focal interactions have strong ecosystem effects; e.g. Schmitz 2004; Fukami *et al.* 2006), these studies typically documented community‐ or ecosystem‐level responses.

Another important goal within the ecosystem perspective has been to understand the linkages between brown (detritus‐based) and green food webs (Allison 2006; Hobbie & Villéger 2015). These connections were traditionally studied from the perspective of plant subsidies to brown food webs, although subsequent empirical work has emphasised the importance of predator control of nutrient cycling and decomposition (Schmitz *et al.* 2010). Likewise, research has focused on intersections between brown and green webs via TTIs associated with detritivore communities. Early studies noted the importance of detrital subsidies to predators (Polis & Strong 1996), and later work has shown that predators of detritivores can influence decomposition rates and plant growth (e.g. Wu *et al.* 2011).

The search for generalities in how trophic interactions affect ecosystem properties has relied on syntheses and meta‐analyses from multiple systems (e.g. Pace *et al.* 1999; Shurin *et al.* 2002; Estes *et al.* 2011; Box 1). These quantitative reviews have demonstrated definitively that top‐down trophic cascades occur (i.e. predator effects on herbivores and plants or algae), and that these are stronger with vertebrate than invertebrate carnivores and with simple than reticulate food web structure (Brett & Goldman 1996; Micheli 1999; Schmitz *et al.* 2000; Halaj & Wise 2001; Mooney *et al.* 2010). Comparisons among ecosystem types – including lakes, streams, forests, grasslands, kelp beds, and marine plankton – have, in turn, shown top‐down trophic cascades to be stronger in aquatic than terrestrial systems, likely due to the faster growth rates and greater palatability of aquatic primary producers (Shurin *et al.* 2002; Shurin *et al.* 2005; Borer *et al.* 2005). Subsequent work comparing the effects of plant fertilisation and predators found stronger top‐down than bottom‐up control, that these effects operated independently, and that these patterns were consistent among marine, freshwater, and terrestrial ecosystems (Borer *et al.* 2006).

## Challenges and Future Directions

As a result of the historic separation of perspectives on TTIs, little is known of how food chain‐level dynamics scale up to drive community‐ and ecosystem‐level processes, or how the latter two feedback to shape food chain‐level TTIs. Here, we outline a conceptual framework that aims to integrate research across levels of biological organisation using tri‐trophic theory as a unifying instrument (Fig. 1b). Beginning with food chain‐level TTIs, we outline gaps in knowledge and future directions to refine existing ecological and evolutionary theory. We then progress through levels of biological organisation, demonstrating how the lens of species interactions theory developed for simple tri‐trophic food chains can be scaled up to communities and ecosystems (Fig. 1b). Finally, we consider how the abiotic contexts within which ecosystems are embedded can feedback to drive both plasticity and evolutionary changes in species traits and thus structure component food chain‐level TTIs (Fig. 1b). At each step, our goal is to consider how TTIs at a one level of organisation emerge from those occurring at lower levels.

### Tri‐trophic food chains

**Figure 2:**
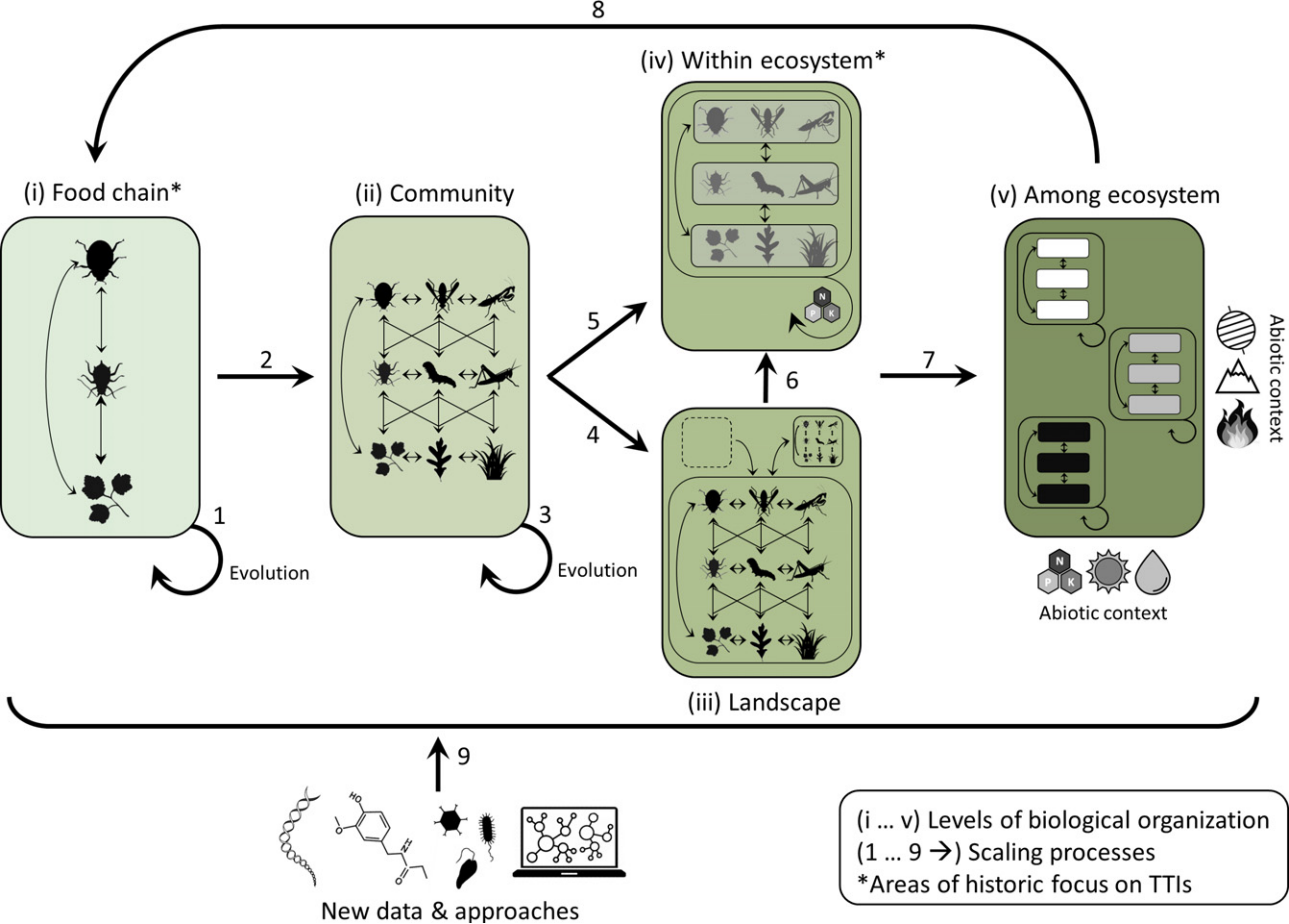
Diagram depicting a conceptual framework for scaling from simple tri‐trophic food chains to cross‐ecosystem variation, and, in turn, how variation in the abiotic environment influences species traits mediating interactions within component food chains. Boxes depict levels of biological organisation spanning from food chains, to local communities, to landscape‐level processes (i.e. processes connecting multiple local communities) and within ecosystem‐level processes (i.e. ecosystem properties emerging from local communities), and finally, to among‐ecosystem variation (i.e. abiotically driven variation in ecosystem properties across different ecosystem types); arrows depict key challenges for scaling up across these levels and in so doing bridge tri‐trophic perspectives. Tri‐trophic research has historically focused on either the food chain level (species interactions perspective) or the within‐ecosystem level (ecosystem perspective). We argue that a key way forward is applying the lens of tri‐trophic theory developed within the species interactions perspective to other levels of organisation to scale up across levels. New technologies and tools in genomics, chemical ecology and microbial ecology will aid in unveiling the mechanistic basis of tri‐trophic food chains as well as linking these component interactions to community structure and ecosystem function.

Despite the historical focus on individual species and their interactions within food chain‐level TTIs (Fig. 2, ‘food chain’), fundamental gaps remain in our understanding of the mechanisms and processes underlying species interactions at this level. Achieving such an understanding of food chains serves as a basis to strengthen theory on tri‐trophic food chains, and, in turn, expand from component TTIs to higher levels of biological complexity.

From an ecological standpoint, there is a vast body of research showing how chemical compounds mediate interactions within plant‐arthropod food chains. These studies have typically involved one species at each of two or more trophic levels under controlled laboratory or greenhouse conditions (Kessler & Heil 2011; Heil 2014), though recent work is shifting towards experimental field conditions (mesocosms or semi‐natural plant communities; Aartsma *et al.* 2017; Kergunteuil *et al.* 2019). This work been highly valuable to understand the mechanistic underpinnings of food chain‐level TTIs. For example, we now recognise that there is a high degree of chemical specificity and complexity involved in TTIs, especially in relation to interactions mediated by plant chemistry (Dicke 2009; Heil 2014; Turlings & Erb 2018). In addition, herbivores and natural enemies can manipulate plant chemistry, including HIPVs, thus linking interactions across all trophic levels (e.g. Musser *et al.* 2002; Sarmento *et al.* 2011; Poelman *et al.* 2012; Zhang *et al.* 2019). Yet, a number of important gaps remain, many of which relate to understanding how the broader community influences component TTIs. For example, the functional role of HIPVs has been studied separately within different ecological settings (Heil 2014; Moreira & Abdala‐Roberts 2019), including plant–plant communication, herbivore deterrence or attraction, indirect defence, and belowground interactions (e.g. mycorrhizal networks and root HIPVs). This separation, however, is unrealistic given that many of these plant compounds probably mediate multiple types of interactions (Heil 2014). For example, relatively little is known about how the direct effects of HIPVs on herbivores (e.g. deterrence) influence their vulnerability to natural enemies by interfering with host location or increasing their detectability, or whether the degree of specificity in natural enemy recruitment to plants mediates interactions among natural enemies (e.g. intra‐guild predation; Janssen *et al.* 1997).

From an evolutionary standpoint, few studies have explicitly tested how natural selection on species traits acts within food chain‐level TTIs (Fig. 2, arrow 1). Changes in species fitness‐correlates due to TTIs have been assumed to imply corresponding evolutionary effects, but there are few studies documenting heritable genetic variation or selection on plant or herbivore traits (e.g. Abrahamson & Weis 1997; Abdala‐Roberts *et al.* 2014). For example, it is often presumed that natural enemies select on plant traits associated with indirect defence, but hard evidence is rare. The few studies testing for such effects have shown that ants select on plant extra‐floral nectary size and nectar production (Rudgers 2004; Rutter & Rausher 2004). Likewise, the adaptive role of producing HIPVs (for plants) or detecting them (for natural enemies) remains largely untested (but see Schuman *et al.* 2012; Kergunteuil *et al.* 2019). For example, work on parasitoid learning and prey location has frequently found low specificity in their responses to HIPVs (but see Giunti *et al.* 2015), suggesting that diverse natural enemies species have evolutionarily converged to respond to a similar suite of plant compounds for the location of herbivores as prey. In the same way, the evolutionary benefits of HIPVs for plants will be greater if this signal is utilised by a wide range of natural enemies. Consistency in plant emissions and enemy responses could, in turn, make it difficult for herbivores to manipulate HIPVs to avoid being found by natural enemies, that is, most HIPV blends are reliable signals of prey presence and are not highly refined such that any disruption in volatile blends is less likely to affect information transfer or content. Also unstudied are the evolutionary effects of natural enemies on plant direct resistance against herbivores (Estes *et al.* 2013). Natural enemies, by reducing herbivory, are expected to dampen selection for plant investment in costly resistance traits, but these evolutionary indirect effects remain largely untested (but see Steinberg *et al.* 1995; Abdala‐Roberts *et al.* 2014). Similarly, many herbivore traits (e.g. diet breadth, toxin sequestration) are presumed to evolve in response to the dual effects of plant defences and natural enemies (see ‘*interactions perspective’* above), but rigorous evolutionary tests are lacking (see Abdala‐Roberts & Mooney 2015).

### Connecting TTIs food chains to communities

There is a fundamental need within the species interactions perspective to place three‐species TTIs in a broader community context. Accordingly, scaling up from food chains composed of single species at each trophic level to community‐level TTIs or food webs (Fig. 2, arrow 2) requires incorporating complexity (e.g. species diversity, composition) at each trophic level (Hunter & Price 1992). In this sense, while there is a substantial body of bi‐trophic work addressing community‐level complexity among pairs of interacting trophic levels, complexity within and among trophic levels has received relatively little attention from a TTIs perspective. At each level, we next describe past work on TTIs that contributes towards this goal while also reviewing pertinent bi‐trophic work and illustrating the gaps in knowledge and new questions that become apparent from applying a tri‐trophic perspective.

Plant community‐level complexity can exert strong effects on plant–herbivore interactions, and in so doing determine the outcome of TTIs. A rich body of research has centred on the influence of plant community structure on herbivorous insects (Agrawal *et al.* 2006; Moreira *et al.* 2016). For example, the composition and diversity of plant species and their secondary chemistry can affect insect herbivores through multiple mechanisms (Moreira *et al.* 2016; Schuman *et al.* 2016), including associational effects (Atsatt & O’Dowd 1976; Barbosa *et al.* 2009; Underwood *et al.* 2014). One of the main conclusions from this work is that herbivore responses to plant community complexity are contingent upon herbivore traits such as mobility and diet breadth; dietary specialist herbivores are usually more strongly (and negatively) affected by plant diversity than generalists (Jactel & Brockerhoff 2007), and plant diversity effects are stronger on mobile than sedentary insect herbivores (Bommarco & Banks 2003). Relatedly, co‐occurring plant species with shared herbivores may interact through apparent competition, which may, in turn, influence plant species composition and coexistence (e.g. Lau & Strauss 2005; Orrock *et al.* 2008). Yet, missing from this bi‐trophic work is an understanding of these phenomena under a tri‐trophic context that incorporates natural enemies. Accordingly, the lens of food chain‐level TTI theory illuminates unstudied questions, such as whether natural enemies mediate plant associational effects or apparent competition through effects on herbivore behaviour. Likewise, despite early work recognising the importance of studying HIPVs in complex environments (Dicke & van Loon 2000), there is still a need to address the function of these compounds within diverse plant communities and how they mediate plant location by herbivores and herbivore location by natural enemies (Heil 2014; Kigathi *et al.* 2019). This is a fundamental gap, since HIPVs are not private information channels only affecting single species of natural enemies, but extend to multiple species at all trophic levels potentially using these traits.

Herbivore community complexity can also affect plant–herbivore and herbivore–natural enemy interactions, and in so doing shape TTIs. With respect to plant–herbivore interactions, research has shown that multiple herbivore species feeding on a common host plant can interact indirectly via plant‐induced responses (Ohgushi 2005), with outcomes for plants and herbivores being contingent upon the herbivore feeding guild; those inducing the same plant defensive signalling pathway (e.g. jasmonic‐ vs. salicylic acid‐mediated responses; Thaler *et al.* 2012) frequently affect each other negatively, whereas herbivore guilds inducing opposing pathways presumably have positive effects due to interference between plant signalling pathways (Thaler *et al.* 2012; but see Moreira *et al.* 2018a). Similarly, a separate body of research has addressed the effects of herbivore community complexity on herbivore–natural enemy interactions. For example, apparent competition among herbivores (i.e. negative indirect interactions through shared natural enemies) is presumed to be an important mechanism mediating species coexistence and community structure (Kaplan & Denno 2007; Holt & Bonsall 2017). Considering these historically bi‐trophic interactions within the context of food‐chain level TTI theory opens novel opportunities and could change the way in which we think about these phenomena. Open questions include whether natural enemies affect the strength or outcome of plant‐mediated herbivore interactions (e.g. De Rijk *et al.* 2016; Blubaugh *et al.* 2018), and whether plant indirect defence traits affect apparent competition among herbivores when the latter share enemies.

Community‐level heterogeneity within natural enemy trophic level may also affect herbivore–natural enemy interactions, with such effects ultimately altering TTIs. Empirical studies on multiple predator effects (MPEs), omnivory and intra‐guild predation (IGP) address non‐additive effects emerging from diverse natural enemy communities (Finke & Denno 2004; Finke & Snyder 2008; Griffin *et al.* 2013). This work has made key contributions to understanding how interactions among natural enemies determine their coexistence and the strength of top‐down control, and how such interactions are influenced by biotic and abiotic factors (Langellotto & Denno 2004; Barton & Schmitz 2009; Sentis *et al.* 2017a). Key findings have been that IGP frequently reduces the strength of natural enemy top‐down control, but that complementary natural enemy traits (e.g. predators or parasitoids feeding in different micro‐habitats or having different foraging mode) can increase top‐down control (e.g. Schmitz 2007). Expanding upon this research to include a tri‐trophic perspective brings novel questions; for instance, little is known of how plant species traits (e.g. HIPVs or plant architecture; Denno *et al.* 2005; Poelman *et al.* 2013) or plant community composition mediate MPEs (i.e. increase or reduce interference between natural enemies or intra‐guild predation), or how herbivores evolve in response to the joint effects of plant defence and MPEs. In addressing this challenge, differentiating between predators and parasitoids will be of key importance because of markedly higher diversity and dietary specialisation observed for parasitoids (Lill *et al.* 2002; Hrček & Godfray 2015), which can influence the outcome of community‐level TTIs.

The consequences of community‐level complexity can also be considered across multiple trophic levels simultaneously. For example, the Enemies Hypothesis (EH; Root 1973) proposes that plant diversity increases natural enemy abundance and diversity, which, in turn, enhances top‐down control of herbivore abundance. The EH has been especially well‐tested and supported in agricultural systems (Russell 1989; Letourneau *et al.* 2011). However, less is known about natural systems or the precise mechanisms by which plant diversity affects natural enemies. Profitable future directions include elucidating which plant traits (or species trait combinations) exert the strongest effects on natural enemy community structure and top‐down control (Stenberg *et al.* 2015). Similarly, the extent to which the EH operates via changes in natural enemy abundance vs. diversity is largely unknown (but see Nell *et al.* 2018). Finally, although it has long been established that plant diversity promotes increased primary productivity (Hooper & Vitousek 1997), the extent to which this pattern is due to greater top‐down control of herbivores as predicted by the EH remains essentially unknown (Haddad *et al.* 2009).

Incorporating trophic complexity into our understanding of community structure and ecosystem processes will be facilitated by a number of emerging quantitative approaches. For example, the application of network theory to interactions between diverse trophic levels has revealed patterns of network structure (e.g. distributions of interactions along specialist to generalist continuum) and their consequences for community stability (Bascompte 2009). Expanding on these analytical approaches to include TTIs (e.g. Wallach *et al.* 2017) can help integrate tri‐trophic theory into each of the previously described bi‐ and multi‐trophic contexts and answer novel questions about the effects of species interactions on community structure. For example, analyses of food web modules (subsets of broader network) can help to tease apart direct and indirect effects from multi‐tropic interactions (Gilman *et al.* 2010). Likewise, Bayesian hierarchical modelling (Arab & Wimp 2013) and mathematical modelling of multi‐species interaction modules (Spiesman & Inouye 2015) can also contribute elucidate the connections between food chain‐level and community‐level TTIs (Barbier & Loreau 2019). Combining these network or food web module analyses with experimental procedures can shed light into how plant–herbivore networks are influenced by natural enemies (in control vs. natural enemy‐excluded treatments) or how herbivore–natural enemy networks are influenced by plant community composition (in plant diversity or composition treatments).

Finally, community‐level TTIs can also affect the evolution of the species embedded within complex food webs (Fig. 2, arrow 3). Evolutionary studies of TTIs have been framed around how herbivore and plant traits evolve within food chain‐level TTIs (see ‘*tri‐trophic food chains’* above; Abdala‐Roberts & Mooney 2015). Accordingly, studies addressing species evolution within the context of complex food webs are needed. For example, predictions for selection on plant resistance to herbivores depend critically on considering the food web in which a plant–herbivore interaction is embedded (Styrsky & Eubanks 2007; Mooney & Agrawal 2008). Potentially fruitful approaches include estimating natural selection within the context of multi‐species interactions (e.g. terHorst *et al.* 2015) and applying network analyses to understand how community structure affects the evolution of component interactions (e.g. Andreazzi *et al.* 2017).

### Connecting communities to meta‐communities and ecosystems

Scaling from community‐ to landscape‐level TTIs (Fig. 2, arrow 4) requires addressing the movement of individuals (plants, herbivores, natural enemies) or resources (e.g. nutrients, detritus) among habitat patches within a community (i.e. meta‐community dynamics) or among different communities (i.e. subsidies) (Polis *et al.* 1997; Taylor *et al.* 2015). Although meta‐community processes are acknowledged as important drivers of community structure (Liebold *et al.* 2004), most of this work has focused on single trophic levels or bi‐trophic interactions, including how features such as vegetation fragmentation and connectivity drive the abundance and distribution of consumers (herbivores and natural enemies) via source‐sink dynamics (Gripenberg & Roslin 2007; Murphy *et al.* 2016). In comparison, relatively little is known about these meta‐community processes in a tri‐trophic context (Guzman *et al.* 2019). Likewise, research on subsidies adopting a tri‐trophic view has focused largely on how movement of resources between terrestrial and aquatic ecosystems boosts predator populations to strengthen trophic cascades (e.g. Nakano & Murakami 2001; Sabo & Power 2002; Spiller *et al.* 2010; Gratton *et al.* 2017), but effects of subsidies on other tri‐trophic phenomena have not been addressed (but see Knight *et al.* 2005). Unstudied questions with respect to subsidies and meta‐community dynamics affecting landscape‐level TTIs include the following: how the degree of mobility varies among trophic levels; whether natural enemies affect herbivore movement, how this varies based upon herbivore traits (e.g. diet breath), and the indirect consequences of such dynamics for plants; and how plant traits or community structure influence movement of natural enemies or herbivores.

Landscape‐level processes scale up to influence ecosystem‐level TTIs (Fig. 2, arrow 6) through the movement of organisms acting as prey, resources or ecosystem engineers (Hastings *et al.* 2007; Taylor *et al.* 2015). To date, however, work measuring ecosystem‐level responses to subsidies has usually not linked these changes to TTIs, and work on meta‐community dynamics has remained largely separate from ecosystem ecology.

Ecosystem‐level TTIs are also an emergent property of community‐level TTIs (Fig. 2, arrow 5). Past work aimed at bridging community‐ and ecosystem‐level TTIs has focused on systems where a diversity of species can be abstracted into a tractable number of guilds that can be modelled as tri‐trophic food webs of relatively few, strong interactions. This approach has been especially successful within aquatic communities, where species and functional diversity may be lower than in most terrestrial counterparts (Strong 1992) as, for example, when rivers or lake ecosystem processes are modelled as four‐level food chains (algae, algivorous zooplankton, planktivorous fish, and piscivorous fish; e.g. Carpenter *et al.* 1987; Power 1990). Results from aquatic studies demonstrate high temporal dynamism in trophic structure with community regulation being alternately driven by nutrients and natural enemies as a function of seasonality in abiotic forcing (Carpenter *et al.* 1987; Power 1990). Within terrestrial ecosystems, Schmitz and colleagues have similarly modelled a meadow community based upon a small number of plant guilds (grasses, dominant herbs in the genus *Solidago* and other herbs), herbivore guilds (grasshoppers and sap‐feeders), and natural enemy guilds (active hunting and sit‐and‐wait spiders) (e.g. Schmitz 2006; Schmitz *et al.* 2010). Such food webs have then studied through trophic manipulations, behavioural observations, and diet analysis to provide linkages between the mechanistic details of community‐level TTIs and ecosystem processes. Results from these grassland studies point to the importance of natural enemy traits and enemy‐induced changes in herbivore traits (e.g. behaviours) on ecosystem processes, such as productivity and nutrient cycling (Schmitz *et al.* 2004).

Despite these advances, significant gaps in knowledge remain, especially for terrestrial arthropod systems. The spatial scale of studies within aquatic and terrestrial vertebrate systems can be large (e.g. multi‐hectare) because of the ability to selectively manipulate (exclude, remove or add) large‐bodied herbivores and predators (e.g. Krebs *et al.* 1995; Palmer *et al.* 2008). Similarly, the lower level of species and functional diversity in such systems results in commonalities among food web structure and thus also in the community‐level TTIs that drive ecosystem‐level properties. In contrast, studies on terrestrial arthropods have been restricted to small‐scaled mesocosms (one to a few m^2^) because of the challenges of selectively manipulating small‐bodied arthropods (Schmitz 2004). This small spatial scale, in turn, makes it difficult to study patchily distributed plant communities or larger‐sized woody plants, or to investigate landscape‐level questions surrounding meta‐population and community dynamics and trophic subsidies. For instance, the many studies of arthropod TTIs within forest ecosystems have been limited to manipulations of saplings (e.g. Marquis & Whelan 1994) or individual tree branches (e.g. Mooney 2007), thus providing inference with respect to individual tree species but not to the larger ecosystem‐level dynamics. In addition, the greater complexity of arthropod food webs makes it uncertain whether there are broad generalities in how community‐level TTIs drive ecosystem‐level properties. The search for such generalities is nascent because of the few systems that have been sufficiently studied at the appropriate scale.

Progress in the study of terrestrial arthropod systems will come from several complementary approaches. Comparisons among experimentally tractable systems can test for commonalities in linkages between community‐level TTIs and ecosystem processes. Elsewhere, non‐experimental approaches will be required. These may include observations of feeding and behavioural relationships (e.g. Nakano & Murakami 2001) in conjunction with diet analysis through the use stable isotopes and DNA barcoding, particularly in highly diverse arthropod communities (e.g. involving parasitoids; Kaartinen *et al.* 2010; Hrček & Godfray 2015), as well as the use of natural experiments based on temporal or spatial variation in community composition and food web structure. Such observational approaches may also be coupled with tractable experimental approaches such as behavioural manipulations through hormone or pheromone application (e.g. Wertheim *et al.* 2006). Finally, future innovations may facilitate experimental manipulations of arthropod trophic groups at large spatial scales (see Lindroth & Raffa 2017), including the development of taxon‐specific insecticides to manipulate entire guilds.

### Connecting within‐ to among‐ecosystem variation and feedbacks to food chains

Ecosystem‐level TTIs can vary due to spatial variation in abiotic factors such as temperature, aridity, nutrients, disturbance and seasonality (Fig. 2, ‘among ecosystem’). Evidence for the importance of abiotic drivers of ecosystem‐level TTIs comes from meta‐analyses (e.g. Shurin *et al.* 2002; Borer *et al.* 2006) and syntheses (e.g. Leroux & Loreau 2015) highlighting differences in the relative strength of bottom‐up and top‐down forcing among ecosystems (Box 1). Beyond these broad‐scale comparisons, however, lies the need to mechanistically explain variation in ecosystem‐level TTIs (Fig. 2, arrow 7), a challenge that is unlikely to be addressed by meta‐analysis due to the limitations of comparing results among studies differing in design and methodology.

The abiotic environment can drive ecosystem‐level TTIs due to ecological effects on species traits, either through trait plasticity or trait filtering. There is a long tradition within ecological theory of attempting to link abiotic factors to community structure and ecosystem processes. Examples include efforts to relate variation in top‐down effects of predators to disturbance and seasonality (Menge & Sutherland 1987), climate warming (Rosenblatt & Schmitz 2016), soil nutrients (Oksanen *et al.* 1981; Borer *et al.* 2006), aridity (e.g. Nelson *et al.* 2019), and habitat fragmentation due to physical features (Gripenberg & Roslin 2007). Multiple factors may, in turn, work together to drive observed global patterns of increased natural enemy pressure at low latitudes or altitudes (Roslin *et al.* 2017). Other work on abiotic forcing has sought to link variation in productivity (Mittlebach *et al.* 2001) or abiotic stress (Bertness & Callaway 1994) with plant community composition and diversity, including effects on plant community traits related to growth and herbivore defence (Rosenthal & Kotanen 1994). While much of this work has focused on changes in spatial variation in species composition through environmental filtering – that is, abiotic (or biotic) effects on the pool of species capable of occurring within a community or ecosystem – similar dynamics might occur through trait plasticity to abiotic factors. Collectively, this research has improved our understanding of abiotic forcing on the composition of individual trophic levels or its effects on interactions among adjacent trophic levels, but we have as yet to fully incorporate a tri‐trophic perspective into such theory. Unanswered questions include, for example, whether variation in energy availability and productivity shaping plant diversity (Grime 1973; Mittlebach *et al.* 2001) mediates natural enemy diversity and top‐down control as predicted by the Enemies Hypothesis (Root 1973), or whether disturbance‐driven variation in top‐down control (Menge *et al.* 1994; Spiller & Schoener 2007) affects herbivore diet breadth as predicted by the Enemy‐Free Space Hypothesis (Bernays 1988; Bernays & Graham 1988).

Variation in abiotic factors can also drive ecosystem‐level TTIs through micro‐ and macro‐evolutionary processes acting at each trophic level. A great deal of work has been conducted on bi‐trophic interactions, especially within the context of plant defence against herbivores. For example, the Resource Availability Hypothesis predicts that nutrient‐poor soils select for plant species that are slow growing and, as a result, less tolerant of herbivore damage and thus invest more in defensive traits (Coley *et al.* 1985; Fine *et al.* 2004; Endara & Coley 2011). Similarly, more consistent, favourable abiotic conditions associated with lower latitudes and elevations are predicted to result in increased herbivory and predation, which, in turn, select for increased plant direct (Anstett *et al.* 2016; Moreira *et al.* 2018b) and indirect (Rodríguez‐Castañeda *et al.* 2016; Moreira *et al.* 2018b) defences. Likewise, increased specialisation of herbivore diet breadth at lower latitudes and elevations (Dyer *et al.* 2007; Rasmann *et al.* 2014) may be driven by increased predation (as predicted by the Enemy‐Free Space Hypothesis; Bernays 1988) and an increase in ecological niches associated with high plant diversity (Ricklefs & Marquis 2012; but see Rasmann *et al.* 2014). There is thus substantial evidence for selection from the abiotic environment for plant, herbivore, and natural enemy traits of relevance to tri‐trophic food chains.

The key challenge will be to bridge the ecological (plasticity, filtering) and evolutionary (micro and macro) effects of abiotic forcing on species traits taking part in component tri‐trophic food chains (Fig. 2, arrow 8), and from there eventually scale up to higher biological complexity as described previously. This can be achieved by testing for convergence in the functioning of multiple food chain‐level TTIs co‐occurring within a community (Fig. 2, arrow 2), and, in turn, addressing the emergent effects of such community‐level processes on ecosystem‐level TTIs (Fig. 2, arrows 4–6). Local or regional abiotic gradients represent powerful tools in this regard, avoiding the confounding influences of historical processes associated with larger spatial scales (e.g. latitudinal gradients). The best example of this comes from syntheses of studies characterising food chain‐level TTIs along elevational gradients (reviewed by Moreira *et al.* 2018b). These syntheses suggest that abiotic stress associated with increasing elevation results in reduced herbivore and natural enemy diversity and abundance and, as a consequence, weaker consumer top‐down pressure. This, in turn, has been proposed to select for reduced overall investment in plant direct and indirect defences (Moreira *et al.* 2018b). Although not yet tested, this causal link between abiotic variation and TTIs would be expected to lead to predictable variation in ecosystem properties through proposed connections between abiotic forcing, plant chemistry, and community‐level TTIs (Burghardt & Schmitz 2015; Hunter 2016).

Although findings from elevational gradients reveal exciting first steps towards linking spatial variation in abiotic forcing and TTIs, key challenges remain. First, patterns of variation in species traits are likely driven not only by the direct influence of the abiotic environment acting on each trophic level but also by indirect effects acting among trophic levels (Rosenblatt & Schmitz 2016). Disentangling these complex effects will require combinations of trophic and environmental manipulative experiments (e.g. Barton & Schmitz 2009; Wu *et al.* 2011; Sentis *et al.* 2017b). Second, trait variation may be due to a combination of phenotypic plasticity and genetic variation, and in both cases this variation may or may not be adaptive. Determining the sources of variation will thus require combining environmental manipulations with reciprocal transplant experiments, possibly complemented by genomic approaches to determine the genetic bases for trait variation, patterns of gene expression, as well as manipulations of relevant traits. Third, studies of environmental gradients must move beyond characterising individual food chains (reviewed by Moreira *et al.* 2018b; Defossez *et al.* 2018; Kergunteuil *et al.* 2019) to explicitly characterise and compare multiple, co‐occurring tri‐trophic food chains. This approach is analogous to other trait‐based approaches (*sensu* McGill *et al.* 2006), but with a simultaneous focus on plants, herbivores, and natural enemies as well as traits relevant to TTIs (e.g. Frenette‐Dessault *et al.* 2013). Such tests for convergence in traits and emergent TTIs among multiple food chains will not only increase the inference of such findings but also provide the basis for scaling up from food chain‐ to community‐level TTIs. Fourth, community‐level convergence among TTI‐relevant traits and the functioning of tri‐trophic food chains must then be linked to variation in ecosystem‐level TTIs. Finally, such studies must be conducted with respect to multiple types of abiotic gradients.

### Mechanistic insight from new technologies in genomics, chemistry and data analysis

Our understanding of the mechanisms underlying TTIs within and across levels of organisation will be accelerated through multiple new technologies, especially as they are applied in the context of experimental manipulations under field conditions and in combination with novel approaches for analysing large, complex datasets (Fig. 2, arrow 9).

New technologies in analytical chemistry now allow for increased resolution and sensitivity in sampling of volatile and non‐volatile compounds produced by plants and animals. For example, methods such as untargeted metabolomics analyses (e.g. Clancy *et al.* 2017; Peters *et al.* 2018) and real‐time measurements with portable chromatographers or nanosensors (Materić *et al.* 2015) are being used to identify the compounds mediating above‐ and belowground component TTIs in natural communities (reviewed by Soler *et al.* 2013; Turlings & Erb 2018). Accordingly, these methods provide detailed information on chemically mediated mechanisms underlying species interactions that can serve as a basis for scaling up to higher levels of organisation, as highlighted by recent work establishing connections between variation in phytochemical composition and arthropod community structure (Schuman *et al.* 2016; Kessler & Kalske 2018). Work revealing the biochemical pathways underlying such traits can, in turn, elucidate their physiological basis and provide opportunities for fine‐scale phenotypic manipulations (Baldwin 2012).

New genomic technologies provide insight into the genetic basis of the phenotypes underlying TTIs. Such techniques are revealing the genetic architecture of traits and the processes underlying their evolution (e.g. Dobler *et al.* 2012; Whiteman & Mooney 2012) while targeted genetic manipulations (e.g. gene knockouts, transformations or genome editing) to alter species phenotypes can provide new insight into the ecological function of those traits (e.g. Degenhardt *et al.* 2009; see Giron *et al.* 2018). The consequences of such species manipulations can then be documented with respect to community‐, landscape‐ and ecosystem‐level TTIs.

Genomics and sequencing techniques are also enabling ecologists to identify groups of microbes or individual taxa of importance to arthropod‐dominated food chains (Pineda *et al.* 2010; Venturi & Keel 2016). This work is revealing how microorganisms manipulate plant and animal phenotypes of relevance to food chain‐level TTIs such as plant‐based volatile compounds (Davis *et al.* 2013; Rasmann *et al.* 2017). Many of these studies have looked at microbial effects on plant–insect bi‐trophic interactions (e.g. plant–herbivore interactions; Shikano *et al.* 2017), but there is ample opportunity for expanding this work to TTIs. This includes free‐living microbes in the rhizosphere (Venturi & Keel 2016), endosymbiotic bacteria in insects (e.g. Rothacher *et al.* 2016; Mclean *et al.* 2017), and endophytic fungi (Van Bael *et al.* 2017), all of which have the potential to affect plant and arthropod traits of relevance to TTIs. Isolating and testing the effects of volatile compounds mediating below‐ and aboveground microbe‐mediated TTIs will be of key importance.

In addition to microbe mediation of plant and animal phenotypes, microbial community ecology is also revealing how trophic interactions among microbes can drive ecosystem function (Allison & Martini 2008; Graham *et al.* 2016). Such studies have been largely based on the manipulation of soil microbiomes or functional groups, but future advances may allow for investigations of TTIs within microbial communities. Metagenomic tools offer an unprecedented opportunity for developing research on tri‐trophic microbial ecology, as well as integrating this with arthropod community ecology and ecosystem ecology.

Finally, advances in remote sensing and ecosystem‐level modelling can help connect TTIs with ecosystem processes. The availability of large databases is increasing not only for remote‐sensed plant diversity and traits (Asner & Martin 2011; Wang & Gamon 2019) but also for global distributions of invertebrates (e.g. ants; Guenard *et al.* 2017). Emerging fields like ecoinformatics can relate trait measurements, species occurrences across trophic levels, landscape‐level processes, and variation in ecosystem function within and among ecosystems (LeBauer *et al.* 2013; Violle *et al.* 2014), therefore contributing to bridge species and ecosystem perspectives.

## Summary and outlook

Knowledge gained from research on TTIs has driven the development of much ecological and evolutionary theory, and empirical work demonstrates their importance for the function of both natural and managed systems. Nevertheless, the lens of tri‐trophic theory has not been applied to all levels of biological organisation. By doing so here, we point out gaps in our understanding and suggest novel ways to form linkages across scales of biological organisation by using TTIs. Our review suggests many novel questions that this proposed programme of research can address, but two key challenges subsume many of these finer points. First, determining whether and how ecosystem‐level TTIs emerge from food chain‐ and community‐level TTIs. Second, determining whether and how ecosystem‐level processes and abiotic factors feedback to shape the species traits that drive TTIs. Addressing these challenges will ultimately unite tri‐trophic perspectives under a single paradigm that guides future research in ecology and evolutionary biology.

## Authorship

LAR and AP wrote the manuscript, KM and CB edited the manuscript, all content is based on discussions that took place during a workshop attended by all authors under the initiative of KM and CB. All co‐authors contributed substantially to revisions.

## Data Availability Statement

No new data are associated with this manuscript.
